# The impact of surgery for vulval cancer upon health‐related quality of life and pelvic floor outcomes during the first year of treatment: a longitudinal, mixed methods study

**DOI:** 10.1002/pon.3992

**Published:** 2015-09-25

**Authors:** Georgina L. Jones, Richard M. Jacques, Joanne Thompson, Hilary J. Wood, Jane Hughes, William Ledger, Mo'iad. Alazzam, Stephen C. Radley, John A. Tidy

**Affiliations:** ^1^ University of SheffieldSection of Health Economics and Decision Science, ScHARRSheffieldUK; ^2^ Design, Trials and StatisticsUniversity of Sheffield, ScHARRSheffieldUK; ^3^ Academic Unit of Primary Medical CareUniversity of SheffieldSheffieldUK; ^4^ University of New South WalesSydneyNSWAustralia; ^5^ UPMC Beacon HospitalDublinIreland; ^6^ Sheffield Teaching Hospital NHS Foundation Trust, Royal Hallamshire HospitalDepartment of Obstetrics and GynaecologySheffieldUK; ^7^ Sheffield Teaching Hospital NHS Foundation Trust, Royal Hallamshire HospitalDepartment of Gynaecological OncologySheffieldUK

## Abstract

**Objective:**

To measure the long‐term impact of surgical treatment for vulval cancer upon health‐related quality of life and pelvic floor outcomes during the first year of therapy.

**Methods:**

Prospective, longitudinal, mixed‐methods study. Twenty‐three women aged >18 years with a new diagnosis of vulval cancer were recruited. The EORTC QLQ C30, SF‐36 and an electronic pelvic floor assessment questionnaire (ePAQ‐PF) were administered at baseline (pre‐treatment) and 3, 6, 9 and 12 months post‐treatment. Mixed effects repeated measures models (all adjusted for age and BMI) were used to investigate changes over time and differences between cancer stage. Qualitative interviews were carried out with 11 of the women and analysed using a thematic approach.

**Results:**

Mean age was 59.9 years (SD = 15.3; range = 23.8–86.6 yrs). Mean BMI was 30.0 (SD = 4.5; range = 24.4–38.2). Sixteen women had early (Stage 1 to 2B), and seven women had advanced stage disease (Stage 3 to 4B). Questionnaire scores revealed that physical and social functioning, fatigue, pain and general sex life were significantly worse at 12 months than pre‐treatment (p = < 0.05). Qualitative analysis revealed multiple treatment side effects which were perceived as severe and enduring. Women with advanced vulval cancer had significantly worse SF‐36 mental health scores at 12 months compared to women with early stage disease (p = 0.037).

**Conclusions:**

Surgery for vulval cancer has long‐term implications which can be persistent 12 months post‐treatment. High rates of morbidity relating to lymphoedema and sexual function re‐enforce the need for specialist clinics to support women who suffer these complications. © 2015 The Authors. *Psycho‐Oncology* published by John Wiley & Sons Ltd.

## Introduction

In the UK, vulval cancer accounts for approximately 8% of gynaecological cancers (Cancer Research UK, 2014) 1. However, numbers are increasing especially in younger women 2. This is linked to increasing incidence of vulval intraepithelial neoplasia (VIN) because of infection with the human papilloma virus (HPV) 3, 4.

Although some women will receive primary radiotherapy, the main treatment is surgery to remove the tumour including a one cm area of healthy tissue 5. The treatment pathway undertaken depends upon several factors. Women with at least stage 1B disease are also offered inguinofemoral lymphadenectomy, if co‐morbidity permits, to assess lymph node involvement. Adjuvant radiotherapy and concomitant chemotherapy may be given if adverse prognostic factors, e.g. close surgical margins or spread to the inguinal lymph nodes are present.

However, despite advances in treatment, morbidity and complications are still high with 40% of women experiencing short‐term complications such as wound breakdown and infection and 28% developing long‐term lymphoedema. Women who have short‐term complications, after groin node dissection, are more likely to develop long‐term complications 6, 7, 8.

A systematic review in 2006 identified no studies which had assessed the impact of treatment upon the health‐related quality of life (HRQoL) of women with vulval cancer 9. More recent reviews conclude that whilst there is still a paucity of research, surgery for vulval cancer creates numerous challenges and impacts upon many areas of quality of life including sexual, psychological and relationship functioning 5, 10. Several qualitative research papers also support these findings 6, 11, 12, 13, 14, 15.

One key limitation of the quantitative data on the HRQoL of women with vulva cancer is that most of the data has been collected post‐treatment, and therefore the change in HRQoL as a consequence of treatment could not be quantified 16, 17, 18, 19, 20, 21, 22, 23, 24. Very little prospective, longitudinal patient‐reported outcome data is available that includes a pre‐treatment baseline assessment 25, 26, 27.

In response to the lack of prospective studies, our aim was to use qualitative and quantitative methods to comprehensively investigate the long‐term impact that surgery for vulva cancer has upon patient outcomes and explore how this may change during the first 12 months from diagnosis to post‐treatment.

## Methods

Women attending the Sheffield Gynaecological Cancer Centre between March 2007 and December 2009 with a new diagnosis of vulval cancer were approached to participate in the study by their clinician. Those interested were given the patient information sheets by HW and asked to return the consent form within one week if willing to participate. We aimed to recruit as many women as possible within this period. With a sample size of 20, 80% power and 5% two‐sided significance, the minimum standardised effect size we could detect is 0.66 (using n = 23, this value would be 0.61).

## Ethical approval

Ethical Approval was obtained from the North Sheffield Ethics Committee (05/Q2308/153).

### Data collection

Demographic data was recorded on a proforma. Clinical data was completed immediately prior to treatment and at subsequent visits. Questionnaires to measure HRQoL and pelvic floor function were administered at baseline (pre‐treatment) and then at 3, 6, 9 and 12 months post‐treatment. It has been argued that because generic measures have been designed to measure HRQoL across a wide variety of conditions, they may not be sensitive enough to assess changes in specific illnesses and therefore a disease specific measure should also be used 28. Therefore, the cancer‐specific European Organisation for Research on Treatment of Cancer (EORTC‐QLQ‐30) 29 and the Short Form‐36 (SF‐36) were administered 30. Because of the nature of the surgery upon the vulva and vagina, we also administered the electronic Personal Assessment Questionnaire‐Pelvic Floor (ePAQ‐PF). It is a web‐based interactive questionnaire which provides an in‐depth evaluation of a woman's pelvic floor symptoms and their impact upon HRQoL 31, 32.

### Statistical analysis

The statistical package for the social sciences (SPSS) for windows, Version 20 (IBM, Armonk, USA) was used. All graphs were plotted using the statistical package R (R Foundation for Statistical Computing, Vienna, Austria).

Questionnaires were scored according to their appropriate scoring algorithms. Changes over time in the domain scores and differences in follow‐up domain scores between early stage (stages 1 and 2 were combined as the cancer was confined to the vulva) and advanced stage cancer (included stage 3 and stage 4) were investigated using mixed effects repeated measures models. Using the approach suggested by Walters 33, three difference models were fitted: (a) HRQoL Domain Score = Time + BMI + Age; (b) HRQoL Outcome = Baseline Score + Time + Stage + Time × Stage Interaction + BMI + Age; (c) HRQoL Outcome = Baseline Score + Time + Stage + BMI + Age.

Model (a) was used to investigate changes in the mean domain scores over time. Any significant effects of time in the model were investigated further using contrasts to compare baseline mean score with mean domain score at each follow‐up time point. Models (b) and (c) were used to investigate difference in follow‐up scores between early and advanced stage cancer adjusting for baseline score. Model (b) has an interaction term between time and stage that allows the effect of stage to be different at each time point. If the interaction term in model (b) was not statistically significant then model (c) was fitted. Model (c) allows for an overall effect of stage that does not chance with time. An AR(1) correlation structure was used in all models to allow for correlation between successive measurements and all models were adjusted for the effect of age and BMI.

Fitting multiple models to the subscales of questionnaires leads to a problem of multiple testing, therefore unadjusted p‐values and confidence intervals have been reported 34. However, caution has been applied when interpreting results, and the size of any statistically significant changes has been compared to a minimally important clinical difference of ten units.

### Qualitative and mixed‐methods analysis

All women consenting to the main questionnaire study were contacted one month before their first 3 month follow‐up visit and asked if they would like to participate in an interview. If consent was obtained, an interview time was set up and written consent obtained.

Of the 23 participants recruited for the main study, 15 women consented to an additional interview (65%). However, 11 of these women were eventually interviewed because one woman died and the interviewer had left the post before completion of the three remaining interviews. Therefore, for pragmatic reasons, we were unable to ensure that data saturation was reached before completion of the interviews.

To maximise diversity, women were purposively interviewed at different follow‐up time points including 3 months (n = 4: stages 1B, 1B, 1B, 4A), 6 months (n = 4: stages 1B, 1B, 3A, 3A), 9 months (n = 2: stages 2, 1B) and 12 months (n = 1: stage 1B). Interviews were conducted within the University of Sheffield's academic unit of reproductive and developmental medicine by a student not involved in the woman's clinical care.

The interview guide was semi‐structured with open questions (see Supplementary Appendix 1). Interviews were tape recorded, digitalised, transcribed and coded by JT using the QSR NVivo 9 Computer‐Assisted Qualitative Data Analysis Software. Data was analysed using a thematic approach outlined by Braun and Clarke 35 utilising a systematic five‐step approach: familiarisation, generating initial codes, searching for themes, reviewing themes, defining and naming themes. GJ, HW, JH and JT collaboratively analysed and discussed emergent themes in order to ensure a consensus was reached. Following analysis of both sets of data, a triangulation protocol was followed whereby the findings of the qualitative data were coded against the quantitative data to explore where there was agreement (convergence), complementarity, silence (i.e. a theme arises from one data set and not another) or dissonance (contradiction) between the study findings 36.

## Results

Twenty‐three women were approached, and all were recruited at baseline. Mean age was 59.9 years (SD = 15.3; range = 23.8–86.6 yrs; median age = 66.1 yrs), and mean BMI was 30.0 (SD = 4.5; range = 24.4–38.2; median BMI = 28.6). Sixteen of the women had early stage (Stage 1B = 15, Stage 2 = 1), and seven women had advanced stage of disease (Stage 3A = 3, 3B = 1, 3C = 1, Stage 4A = 1, 4B = 1). All of the women were of British nationality, 22 were white Caucasian and 1 woman was of mixed race. Ten (43.5%) were married, 3(13%) cohabiting, 2(8.7%) single, 5(21.7%) widowed and 3 (13%) other. Unfortunately, one woman died before her 3 month follow‐up, and two patients withdrew from the study and had insufficient data to be included in the analysis; therefore 20 women are included in the tables. These two women had also not given consent to an interview.

Eighteen women underwent triple incision vulvectomy (including a bilateral inguinofemoral lymphadectomy). Four women underwent radical wide local excision of the vulva and unilateral groin node dissection. Six women received adjuvant radiotherapy with three of these women also receiving concomitant chemotherapy (Cisplatin). One woman with stage 4 disease received chemo‐radiotherapy but died from her disease.

The longitudinal model for the EORTC showed a significant change over time for physical functioning (p = 0.010), social functioning (p = 0.049), fatigue (p = 0.011) and pain (p = 0.036) (Supplemental Appendix 2).

Post‐hoc comparisons of each time point with baseline showed a significant change (indicating a worse HRQoL) in all these domains from baseline to 3 months (physical functioning = −14.0, 95% CI: −22.2 to −5.8, p = 0.001; social functioning = −19.2, 95% CI: −32.2 to −6.1, p = 0.005; pain = 17.5, 95% CI: 5.2 to 29.8, p = 0.006; and fatigue = 17.8, 95% CI: 8.3 to 27.2, p < 0.001). As these mean differences are greater than the clinically important difference of −10 but the confidence interval includes −10, this change is potentially clinically important (Supplemental Appendix 3). No other statistically significant reductions were observed for pain and social functioning from baseline to 6, 9 and 12 months. However, a significantly worse HRQoL in physical functioning was also observed from baseline to 9 months (−14.8, 95% CI: −26.6 to −2.9, p = 0.0015), and 12 months (−15.0, 95% CI: −27.5 to −2.5, p = 0.019) and from baseline to 6 months only for fatigue (13.9, 95% CI: 2.4 to 25.4, p = 0.019). As these mean differences were greater than the clinically important difference of −10 but the confidence interval includes −10, this change is also potentially clinically important (Supplementary Appendix 3).

On the SF‐36, the longitudinal model showed significant declines in physical functioning (p = 0.003) (Supplemental Appendix 2). Post‐hoc comparisons of each time point with baseline show a significant reduction in physical functioning from baseline to 3 months (−14.4, 95% CI: −22.6 to −6.2, p = 0.001). As the mean difference is greater than the clinically important difference of −10 but the confidence interval includes −10, this change is potentially clinically important. Changes from baseline to 6 months (−10.2, 95% CI: −21.3 to 1.0, p = 0.073), 9 months (−3.3, 95% CI: −16.3 to 9.6, p = 0.613) and 12 months (−10.3, 95% CI: −24.5 to 4.0, p = 0.144) were not statistically significant.

Using ePAQ‐PF the longitudinal model showed an overall significant deterioration over time (p = 0.047) for the general sex life domain (Supplemental Appendix 4). However, post‐hoc comparisons of each time point with baseline showed no significant difference in general sex life from baseline to 3 months (1.8, 95% CI: −6.6 to 10.3), 6 months (−3.7, 95% CI: −14.1 to 6.6, p = 0.470), 9 months (−9.3, 95% CI −21.6 to 32.1, p = 0.137) and 12 months (2.7, 95% CI: −11.0 to 16.3, p = 0.698).

Adjusting for baseline, age and BMI we found that women with advanced compared to early stage vulval cancer had worse mental health at follow‐up as measured on the SF‐36 (−19.3; 95% CI: −37.2 to −1.3; p = 0.037). However, no other significant differences were observed (Table 1).

**Table 1:** Estimated regression coefficients from mixed effects repeated measures models to show the effect of cancer stage on outcome

	Time × cancer stage interaction P‐value	Cancer stage		
		Model coefficient^a^	95% CI	P‐value
EORTC dimension				
HRQoL/global health	0.823	−13.4	(−30.9, 4.0)	0.124
Physical function	0.761	−5.2	(−18.4, 8.0)	0.420
Role function	0.598	−1.1	(−22.6, 20.3)	0.912
Emotional function	0.764	−10.5	(−34.3, 13.2)	0.362
Cognitive function	0.790	0.9	(−15.9, 17.7)	0.912
Social function	0.729	−4.4	(−16.2, 25.0)	0.661
Fatigue	0.472	5.0	(−10.8, 20.9)	0.513
Nausea	0.878	−4.6	(−13.3, 4.1)	0.283
Pain	0.663	−14.2	(−38.8, 10.4)	0.240
Dyspnoea	0.222	2.1	(18.8, 14.5)	0.791
Insomnia	0.406	7.8	(−14.0, 29.6)	0.462
Appetite loss	0.847	11.7	(−11.9, 35.4)	0.320
Constipation	0.891	−6.9	(−4.9, 18.6)	0.242
Diarrhoea	0.916	0.8	(−9.4, 11.0)	0.873
Financial	0.216	7.7	(−5.2, 20.5)	0.224
SF36 dimension				
Physical functioning	0.559	−2.2	(−17.7, 13.2)	0.765
Role physical	0.248	3.6	(−31.9, 39.2)	0.832
Bodily pain	0.227	−4.6	(−25.0, 15.9)	0.644
General health	0.083	4.1	(−8.8, 16.9)	0.518
Vitality	0.482	−0.8	(−19.3, 17.7)	0.930
Social functioning	0.821	−12.4	(−34.4, 9.7)	0.253
Role emotional	0.659	−15.7	(−44.7, 13.3)	0.266
Mental health	0.053	−19.3	(−37.2, −1.3)	0.037
EPAQ dimension				
Urinary pain	0.930	5.9	(−1.9. 13.8)	0.127
Voiding	0.919	−4.5	(−10.7, 1.6)	0.139
Overactive bladder	0.473	4.4	(−8.5, 17.4)	0.974
Stress incontinence	0.603	−0.2	(−9.4, 9.0)	0.962
Urinary HRQoL	0.996	2.9	(−9.9, 15.7)	0.632
Irritable bowel	0.999	−1.0	(−9.0, 6.9)	0.785
Constipation	0.714	−3.2	(−17.5, 11.1)	0.643
Evacuation	0.329	−3.2	(−8.4, 2.1)	0.221
Bowel continence	0.196	2.2	(−4.3, 8.7)	0.491
Bowel HRQoL	0.625	−1.1	(−10.1, 7.8)	0.789
Vaginal pain and sensation	0.927	−6.6	(−22.0, 8.9)	0.379
Vaginal capacity	0.283	−5.9	(33.5, 21.8)	0.654
Prolapse	0.951	−0.9	(−4.3, 2.4)	0.524
Vaginal HRQoL	0.543	−5.0	(−16.4, 6.4)	0.356
Sexual urinary	0.490	−2.6	(−16.0, 10.8)	0.660
Sexual vaginal	0.482	−14.2	(−35.2, 6.8)	0.154
Dyspareunia	0.265	−0.5	(−41.3 40.3)	0.962
General sex life	0.208	−1.5	(−16.0, 13.1)	0.825

a Coefficient for stage from repeated measures model, positive value indicates higher score.

### Qualitative and mixed‐methods results

Twenty overarching themes emerged from the qualitative analysis reflecting the wide impact treatment for vulval cancer had upon the women's HRQoL over the first year, including: physical functioning, ability to carry out daily activities, pain, discomfort/tenderness, lymphoedema, fatigue, social functioning, confidence, impact on work, sexual functioning, emotional well‐being, patient education, patient information, self‐identity and femininity, body image, coping strategies, experiences of in‐hospital care, relationships with their partner, family and children.

Questionnaire scores revealed significantly worse physical, social and sexual function, fatigue, pain and mental health at follow‐up. The results of the triangulation process with these domains are reported in Supplemental Appendix 5. The qualitative data confirmed that all these areas of HRQoL were affected as a result of vulval cancer treatment suggesting a good level of agreement between the datasets. However, a large number of sub‐themes that emerged from the qualitative analysis provided deeper insight and complementary data as to why these questionnaire domains were negatively affected.

Physical functioning (in particular the ability to carry out daily activities and walking) appeared most affected by lymphoedema (particularly leg swelling) and urinary incontinence. Mobility was also affected by the use of drains and numbness/loss of sensation post‐surgery, particularly in relation to going to the toilet. For some women this resulted in the need for help with physical movements and/or inactivity. Pain had a big impact on all women; in most instances this resulted from the site of surgery and subsequent infection and stitches. Overall, pain was described as excruciating (although discomfort/tenderness was also mentioned) with many women relying on painkillers up to at least 6 months post‐treatment. However, for some women pain was also the consequence of lymphoedema and radiotherapy (where administered) with women describing their skin as burnt or blistered, similar to sunburn. Pain impacted upon many areas of physical and sexual functioning and emotional wellbeing.

Most women described feeling very tired and lethargic. This limited their ability to carry out simple physical tasks (e.g. walking, standing), domestic chores at home and work activities. Women often reduced their hours/days and/or adapted their work routine to accommodate the extra fatigue. Some women did report improvements in their energy levels, 6 months post‐treatment.

In relation to social functioning, relationships with family and children and leisure activities were affected. Many women stopped their leisure activities for a short period of time or avoided certain social situations. This appeared to affect their confidence; incontinence and the need to be near toilets were important issues for many women. Sexual functioning was particularly affected; feeling worried and frightened, a loss of interest/avoidance and lack of enjoyment in sex post‐treatment were often reported. Overall, the women reported feeling nervous and anxious about numerous other areas of their life associated with their treatment including washing their vulva, viewing and touching their vulva, fear of cancer recurrence and their own mortality. As a consequence, a range of emotions were often described including feeling withdrawn and depressed, mood swings and frustration. However, despite experiencing such severe side effects there was significant evidence of resilience and positivity in the data which helped patient's cope with their situation.

All these themes appeared present across the dataset. However, improved energy levels/reduced tiredness and fear of cancer recurrence were more salient in women six months post‐treatment. The side effects of radiotherapy were reported in women with more advanced stage disease.

## Discussion

We aimed to use mixed methods to comprehensively understand the impact that vulval cancer surgery has upon patient outcomes and explore how this may change from diagnosis and over the first year post‐treatment. To our knowledge, this is the first mixed‐methods paper in this area. Most of our patient sample underwent major surgery, the most common procedure being a triple incision vulvectomy (including a bilateral inguinofemoral lymphadectomy).

Our analysis revealed that following surgery, women experience significant side effects; pain and fatigue in particular are typically unresolved at 12 months post‐treatment and worse than at pre‐treatment. There were also key areas of HRQoL that significantly deteriorated over time (physical, social and sexual functioning) that were also unresolved at 12 months.

Overall, good convergence was observed following the triangulation process between the quantitative and qualitative data. No silent or dissonant themes emerged which suggested that the questionnaires measure the relevant areas of HRQoL. However, they lack depth as the qualitative analysis revealed many complementary themes which provided a deeper understanding for the quantitative results.

The significant negative impact upon physical functioning was identified by both the EORTC QLQ‐C30 and the SF‐36. Our findings are similar to a previous study 25 whereby, in women who underwent inguinofemoral lymphadenectomy, worse physical and role functioning were reported at 6 months after surgery compared to baseline.

Symptoms most attributed to the problems in ‘physical functioning’ appeared to be pain and lymphoedema. Lymphoedema is a well reported side effect of surgery for vulval cancer following groin dissection 37. de Melo and colleagues 21 also observed a significant correlation between lymphoedema of the lower extremities (LLE) and worse physical functioning. Whilst Sentinel node biopsy may result in less treatment‐related lymphoedema 22, 24, unfortunately it is not available to patients at our institution.

As well as describing how lymphoedema impacted upon mobility, women also described it as causing numbness, pain and tiredness. These side effects impacted upon psychological and social functioning which is supported by other studies 37, 38. On the EORTC, we found pain and fatigue got significantly worse over time. Novackova et al. 30 also observed more fatigue 6 months after surgery compared to baseline. Although, it is most likely that lymphoedema was a main contributor to these outcomes, from the qualitative analysis, women described other side effects such as wound infections, drains and also skin blistering/burning from the radiotherapy that caused pain and contributed to these negative HRQoL outcomes. Overall, the women perceived these side effects as severe and enduring, affecting social functioning, their confidence and emotional health which is similar to other qualitative literature in this area 11, 15. Mental health was observed to be significantly worse in women with advanced compared to early stage disease. This is perhaps not surprising given the prognosis that women with advanced stage vulval cancer face and additional psychological support may be of benefit for this group of women.

The negative impact that vulval cancer surgery has upon the sexual functioning of women has been well described 10, 16. We found a significant difference on the general sex life domain of the e‐PAQ‐PF which suggests that there was a negative change over time. A high number of our sample had also undergone a lymphadenectomy which has also been reported elsewhere as having a significant negative impact on sexual function 24. A recent study reported no significant differences in psychosocial and sexual functioning for women with vulvar cancer before and after vulvectomy 12 months post‐treatment. However, a different set of questionnaires were used in that study which may explain the findings 27.

On the ePAQ‐PF, domains can be skipped if it is not relevant to the patient. A high proportion of the women had skipped the sex domain questions. One reason for this may be because they did not have a partner as suggested by the demographic data (only 13/23 women reported having a partner). Alternatively, women may not have resumed their sex life (regardless of their relationship status) because of the impact of the surgery. Indeed, the latter was supported from the qualitative analysis which revealed that pain from surgery had prevented most women from engaging in penetrative sex at least up to six months post‐treatment and made the use of dilators difficult and challenging.

There are a few limitations to the study. Whilst we achieved very low loss to follow‐up rates, as with most vulval cancer studies, it is a small study and so may not have established if women with more advanced stage disease do have greater physical morbidity. It is also a single institution study and so might be biassed as a result. However, it is a non‐selected group of women going through treatment and therefore may reflect the average outcome of care compared with a clinical trial. Despite a particularly young patient in the study, the median age and BMI of the patients are also consistent with the sample of women who usually present with vulval cancer. There were many themes that resonated with all women in the study. However, because just over half of the sample were interviewed, it is possible that the views presented do not extend to other women in the sample (on which the quantitative findings are based). Also, because of the aforementioned pragmatic reasons data saturation cannot be assumed which is another potential limitation to the present findings.

## Conclusion

We found that women who undergo treatment for vulval cancer report high rates of treatment‐related morbidity. The impact of surgery appeared to most negatively affect physical and social functioning and increase fatigue and pain post‐treatment. Recovery from treatment can be a slow process and surgery has long‐term implications. Issues relating to morbidity are widely ignored during the treatment phase and most patient information resources are not explicit in conveying these risks. This study will allow more accurate information to be provided to future patients. Better management of treatment side effects (particularly pain and fatigue) may lead to improvements in HRQoL which need to be monitored and managed closely throughout at least the first year from diagnosis. The high rates of morbidity relating to lymphoedema and sexual function re‐enforce the need for specialist clinics to support women who suffer these complications.

## Author contributions

All named authors have read the manuscript, agreed to the submission and participated in the study to a sufficient extent to be named as authors.

## Conflict of interest

None except Stephen Radley is a director and shareholder in EPAQ Systems Ltd, an NHS spin‐out technology company.

## Supporting information

Additional supporting information may be found in the online version of this article at the publisher's web site.

Supporting info item

Supporting info item

Supporting info item

Supporting info item

Supporting info item
