# Supplementary material for: The impact of surgery for vulval cancer upon health‐related quality of life and pelvic floor outcomes during the first year of treatment: a longitudinal, mixed methods study
Source: Psychooncology. 2015 Sep 25;25(6):656–62. doi: 10.1002/pon.3992 (PMC5054883; doi:10.1002/pon.3992)
Supplement: Supplementary file 1 — Supporting info item [file PON-25-656-s001.docx]

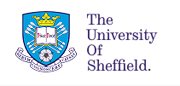

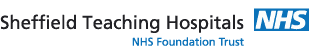


Study Number: STH14587

Study Title: “Treatment for vulval malignancy: measuring outcome

from the patient’s perspective.”

**General Introduction**

Thank you for coming along to talk to us today and helping us with our research. We are interested in understanding and exploring the ways in which the treatment you have received has affected your quality of life. We are hoping that by talking to you today and the other women participating in this study, it will help to inform future patients about the possible impact of therapeutic interventions and provide information of importance to both patients and clinicians in planning treatments.

**Area to Explore/Prompts**

1) Background to the cancer type and treatment(s) received

**Including trajectory of cancer, treatment journey, current situation**

2) General positive and negative impacts

3) Affect on self, including the sense of self as woman, body image – ways of adapting

4) Affect on sex life – ways of adapting

5) Affect on relationship(s) – ways of adapting

6) Possible informational needs – type of information received and the quality of this information? Recommendations regarding future information/support/intervention required

7) Future concerns

8) Other issues participant feels important.
