# Supplementary material for: The impact of surgery for vulval cancer upon health‐related quality of life and pelvic floor outcomes during the first year of treatment: a longitudinal, mixed methods study
Source: Psychooncology. 2015 Sep 25;25(6):656–62. doi: 10.1002/pon.3992 (PMC5054883; doi:10.1002/pon.3992)
Supplement: Supplementary file 2 — Supporting info item [file PON-25-656-s002.docx]

Supplemental Appendix 2: Summary statistics for domains of the EORTC and SF-36 questionnaires

|  | Baseline | | | 3 Months | | | 6 Months | | | 9 Months | | | 12 Months | | | P-Value^1^ |
| --- | --- | --- | --- | --- | --- | --- | --- | --- | --- | --- | --- | --- | --- | --- | --- | --- |
|  | N | Mean | SD | N | Mean | SD | N | Mean | SD | N | Mean | SD | N | Mean | SD |  |
| EORTC |  |  |  |  |  |  |  |  |  |  |  |  |  |  |  |  |
| HRQoL/Global Health | 20 | 66.7 | 20.2 | 20 | 57.1 | 23.6 | 20 | 61.7 | 24.8 | 19 | 59.6 | 28.2 | 20 | 60.0 | 23.5 | 0.203 |
| Physical Function | 20 | 81.0 | 21.0 | 20 | 67.0 | 26.2 | 20 | 71.0 | 24.7 | 19 | 65.3 | 23.9 | 20 | 66.0 | 20.6 | 0.010 |
| Role Function | 20 | 82.5 | 29.4 | 20 | 67.5 | 29.4 | 20 | 75.0 | 25.6 | 19 | 71.1 | 30.3 | 20 | 30.0 | 29.9 | 0.083 |
| Emotional Function | 20 | 70.0 | 26.1 | 20 | 67.1 | 25.1 | 20 | 74.2 | 26.3 | 19 | 67.1 | 29.1 | 20 | 70.8 | 30.3 | 0.564 |
| Cognitive Function | 20 | 78.3 | 26.0 | 20 | 80.0 | 20.7 | 20 | 85.0 | 15.2 | 19 | 81.6 | 21.4 | 20 | 84.2 | 23.9 | 0.742 |
| Social Function | 20 | 88.3 | 25.4 | 20 | 69.2 | 26.1 | 20 | 78.3 | 26.5 | 19 | 72.8 | 33.4 | 20 | 79.2 | 30.0 | 0.049 |
| Fatigue | 20 | 20.6 | 21.7 | 20 | 38.3 | 28.0 | 20 | 34.4 | 23.9 | 19 | 32.2 | 23.1 | 20 | 32.8 | 24.8 | 0.011 |
| Nausea | 20 | 5.0 | 13.4 | 20 | 10.0 | 15.7 | 20 | 7.5 | 12.7 | 19 | 7.9 | 16.1 | 20 | 9.2 | 19.8 | 0.755 |
| Pain | 20 | 15.8 | 31.3 | 20 | 33.3 | 28.1 | 20 | 25.0 | 30.3 | 19 | 33.3 | 33.3 | 20 | 33.3 | 34.6 | 0.036 |
| Dyspnoea | 20 | 20.0 | 27.4 | 20 | 20.0 | 25.1 | 20 | 13.3 | 22.7 | 19 | 26.3 | 28.5 | 20 | 20.0 | 27.4 | 0.063 |
| Insomnia | 20 | 40.0 | 38.4 | 20 | 46.7 | 33.2 | 20 | 40.0 | 31.7 | 19 | 45.6 | 35.5 | 20 | 38.3 | 32.9 | 0.702 |
| Appetite Loss | 20 | 15.0 | 22.9 | 19 | 12.3 | 22.8 | 20 | 13.3 | 22.7 | 19 | 14.0 | 23.1 | 20 | 16.7 | 25.4 | 0.909 |
| Constipation | 20 | 15.0 | 29.6 | 20 | 13.3 | 22.7 | 20 | 13.3 | 22.7 | 19 | 10.5 | 19.4 | 20 | 6.7 | 17.4 | 0.789 |
| Diarrhoea | 20 | 5.0 | 12.2 | 20 | 11.7 | 19.6 | 20 | 1.7 | 7.5 | 19 | 10.5 | 19.4 | 20 | 3.3 | 10.3 | 0.060 |
| Financial Problems | 20 | 3.3 | 14.9 | 20 | 15.0 | 25.3 | 20 | 10.0 | 19.0 | 19 | 10.5 | 15.9 | 20 | 6.7 | 17.4 | 0.067 |
| SF36 |  |  |  |  |  |  |  |  |  |  |  |  |  |  |  |  |
| Physical Functioning | 20 | 60.8 | 31.8 | 20 | 52.3 | 32.6 | 19 | 57.9 | 32.5 | 19 | 62.9 | 30.9 | 20 | 56.5 | 33.6 | 0.003 |
| Role Physical | 20 | 58.8 | 48.2 | 20 | 33.8 | 46.1 | 19 | 39.5 | 48.1 | 19 | 47.4 | 44.0 | 20 | 51.3 | 48.3 | 0.122 |
| Bodily Pain | 20 | 67.8 | 30.0 | 20 | 60.5 | 28.4 | 19 | 68.2 | 30.4 | 19 | 59.2 | 33.6 | 20 | 62.7 | 29.7 | 0.345 |
| General Health | 20 | 66.7 | 20.5 | 20 | 61.0 | 24.2 | 19 | 62.8 | 24.2 | 19 | 59.0 | 27.0 | 20 | 57.9 | 24.6 | 0.495 |
| Vitality | 20 | 55.3 | 20.4 | 18 | 46.6 | 24.7 | 19 | 55.0 | 24.4 | 19 | 51.8 | 26.6 | 20 | 49.5 | 27.0 | 0.178 |
| Social Functioning | 20 | 72.5 | 28.3 | 20 | 62.5 | 30.1 | 19 | 69.7 | 28.4 | 19 | 63.8 | 35.3 | 20 | 61.9 | 34.0 | 0.346 |
| Role Emotional | 20 | 58.3 | 49.4 | 20 | 48.3 | 48.9 | 19 | 57.9 | 48.2 | 19 | 56.1 | 43.1 | 20 | 58.3 | 45.7 | 0.789 |
| Mental Health | 20 | 64.8 | 23.7 | 20 | 65.4 | 24.1 | 19 | 73.2 | 18.5 | 19 | 66.5 | 23.7 | 20 | 69.0 | 20.4 | 0.779 |

^1^P-Value for time from the longitudinal model, a statistically significant results indicates a change in the mean domain score over time.

The EORTC-QLQ 30 is scored between 0 – 100. For the functional scales and the global health status question, a higher score represents a higher/better level of functioning. However, a higher score on the symptom scales indicates a higher/worse level of symptoms. an outcome measure in clinical settings [37].

The SF-36 is scored on a scale whereby 0 = worst health and 100 = best health.
