# Supplementary material for: The impact of surgery for vulval cancer upon health‐related quality of life and pelvic floor outcomes during the first year of treatment: a longitudinal, mixed methods study
Source: Psychooncology. 2015 Sep 25;25(6):656–62. doi: 10.1002/pon.3992 (PMC5054883; doi:10.1002/pon.3992)

Supplemental Appendix 3: Mean change and 95% confidence intervals in key domain scores. Red dashed lines show a clinically meaningful change.


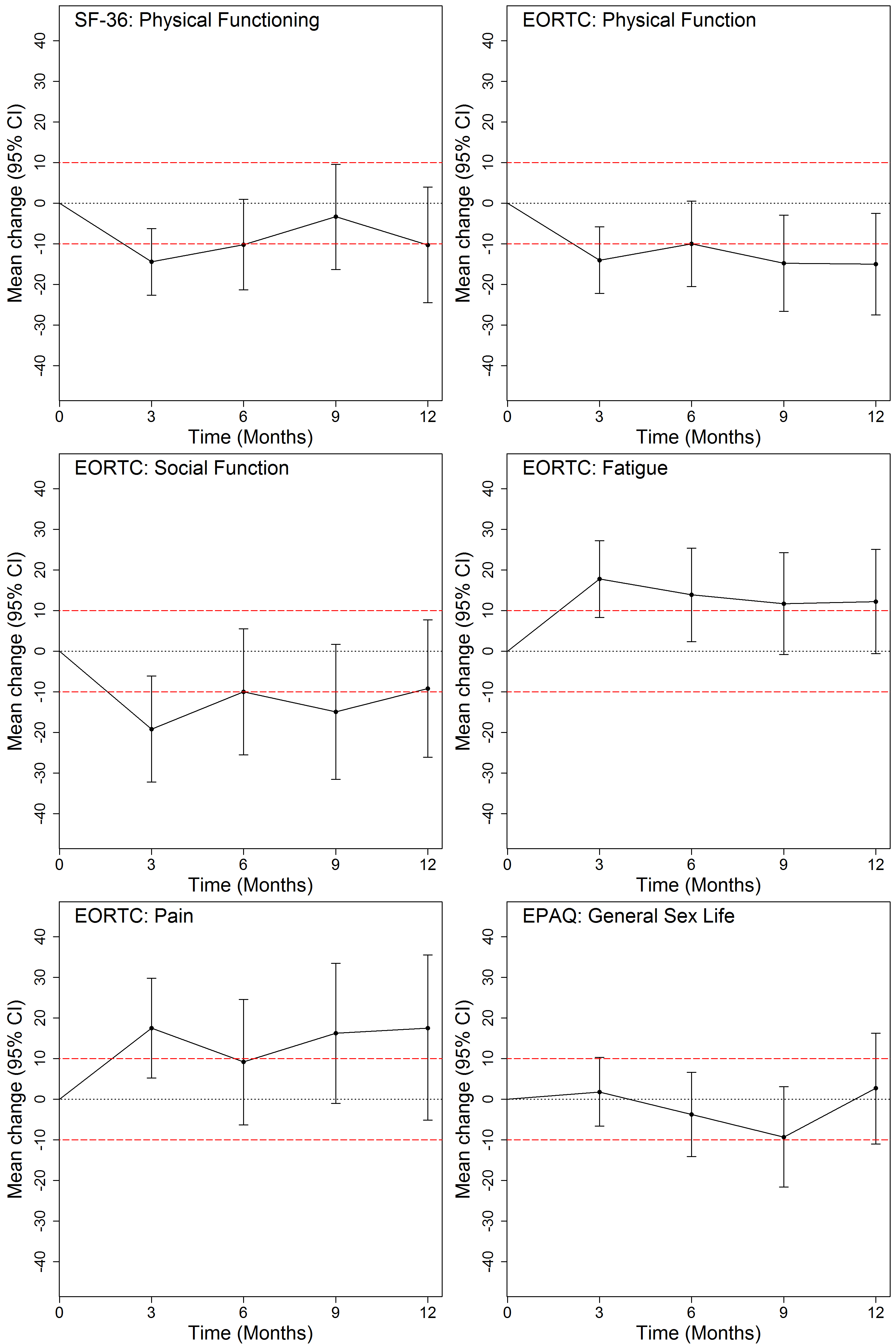

Supplement: Supplementary file 3 — Supporting info item [file PON-25-656-s003.docx]
