# Supplementary material for: The impact of surgery for vulval cancer upon health‐related quality of life and pelvic floor outcomes during the first year of treatment: a longitudinal, mixed methods study
Source: Psychooncology. 2015 Sep 25;25(6):656–62. doi: 10.1002/pon.3992 (PMC5054883; doi:10.1002/pon.3992)
Supplement: Supplementary file 4 — Supporting info item [file PON-25-656-s004.docx]

Supplemental Appendix 4: Summary statistics for domains of the EPAQ questionnaire

|  | Baseline | | | 3 Months | | | 6 Months | | | 9 Months | | | 12 Months | | | P-Value^1^ |
| --- | --- | --- | --- | --- | --- | --- | --- | --- | --- | --- | --- | --- | --- | --- | --- | --- |
|  | N | Mean | SD | N | Mean | SD | N | Mean | SD | N | Mean | SD | N | Mean | SD |  |
| Urinary Pain | 20 | 5.5 | 12.6 | 17 | 2.6 | 6.2 | 16 | 4.8 | 11.3 | 16 | 4.8 | 9.8 | 16 | 5.5 | 12.1 | 0.907 |
| Voiding | 20 | 2.5 | 8.1 | 17 | 1.5 | 6.1 | 12 | 6.9 | 16.9 | 16 | 3.6 | 12.5 | 17 | 2.4 | 8.1 | 0.887 |
| Overactive Bladder | 20 | 12.5 | 16.7 | 17 | 18.6 | 19.4 | 16 | 12.4 | 14.6 | 16 | 17.1 | 15.0 | 17 | 17.1 | 21.8 | 0.219 |
| Stress Incontinence | 20 | 8.7 | 16.2 | 17 | 5.1 | 8.4 | 16 | 7.5 | 12.4 | 16 | 11.3 | 16.0 | 13 | 14.5 | 15.0 | 0.614 |
| Urinary HRQoL | 20 | 6.1 | 12.1 | 17 | 14.3 | 21.3 | 16 | 9.7 | 17.2 | 16 | 13.8 | 18.8 | 17 | 14.4 | 20.0 | 0.262 |
| Irritable Bowel | 20 | 10.0 | 12.7 | 16 | 10.4 | 15.9 | 16 | 8.8 | 14.0 | 16 | 10.0 | 13.3 | 17 | 7.8 | 10.6 | 0.857 |
| Constipation | 20 | 13.3 | 20.2 | 17 | 13.7 | 18.6 | 16 | 8.3 | 16.3 | 16 | 8.9 | 16.2 | 17 | 7.8 | 16.6 | 0.594 |
| Evacuation | 20 | 9.7 | 14.1 | 17 | 7.8 | 10.9 | 16 | 4.3 | 9.1 | 16 | 4.0 | 5.7 | 17 | 4.8 | 10.1 | 0.224 |
| Bowel Continence | 20 | 1.5 | 2.9 | 17 | 3.7 | 8.1 | 16 | 2.4 | 6.4 | 16 | 2.1 | 7.3 | 17 | 3.7 | 8.3 | 0.642 |
| Bowel HRQoL | 20 | 1.1 | 3.4 | 16 | 4.8 | 9.8 | 16 | 2.8 | 8.5 | 16 | 5.6 | 17.3 | 17 | 4.6 | 13.7 | 0.222 |
| Vaginal Pain and Sensation | 19 | 15.1 | 21.2 | 17 | 9.3 | 16.2 | 16 | 8.8 | 19.7 | 15 | 9.1 | 21.9 | 16 | 11.4 | 22.3 | 0.470 |
| Vaginal Capacity | 17 | 3.2 | 10.8 | 15 | 0.7 | 2.8 | 15 | 9.6 | 24.1 | 14 | 11.1 | 27.2 | 14 | 9.5 | 22.2 | 0.351 |
| Prolapse | 20 | 3.2 | 7.4 | 16 | 1.6 | 6.3 | 16 | 0.0 | 0.0 | 15 | 0.7 | 2.8 | 16 | 1.1 | 4.3 | 0.438 |
| Vaginal HRQoL | 20 | 7.8 | 19.1 | 17 | 8.5 | 21.3 | 16 | 5.6 | 19.5 | 16 | 2.8 | 8.5 | 16 | 1.4 | 5.5 | 0.398 |
| Sexual Urinary | 14 | 2.4 | 5.1 | 11 | 0.7 | 2.4 | 13 | 1.3 | 4.7 | 14 | 2.4 | 6.2 | 12 | 4.2 | 12.1 | 0.479 |
| Sexual Bowel | 14 | 0.0 | 0.0 | 11 | 0.0 | 0.0 | 13 | 0.0 | 0.0 | 14 | 0.0 | 0.0 | 12 | 0.0 | 0.0 | - |
| Sexual Vaginal | 13 | 16.2 | 32.8 | 12 | 12.5 | 30.1 | 13 | 9.0 | 25.9 | 13 | 8.3 | 21.2 | 12 | 15.9 | 27.9 | 0.447 |
| Dyspareunia | 8 | 15.0 | 21.8 | 10 | 8.7 | 18.4 | 13 | 8.2 | 21.5 | 13 | 9.8 | 24.5 | 12 | 10.6 | 18.1 | 0.822 |
| General Sex Life | 14 | 18.4 | 29.2 | 13 | 21.8 | 26.7 | 14 | 10.7 | 21.3 | 12 | 13.3 | 23.0 | 12 | 23.6 | 37.4 | 0.047 |

^1^P-Value for time from the longitudinal model, a statistically significant results indicates a change in the mean domain score over time.

The ePAQ-PF is scored on a scale whereby 0 = good health and 100 = worst health.
