# Supplementary material for: The impact of surgery for vulval cancer upon health‐related quality of life and pelvic floor outcomes during the first year of treatment: a longitudinal, mixed methods study
Source: Psychooncology. 2015 Sep 25;25(6):656–62. doi: 10.1002/pon.3992 (PMC5054883; doi:10.1002/pon.3992)
Supplement: Supplementary file 5 — Supporting info item [file PON-25-656-s005.docx]

Supplemental Appendix 5: Results of the Triangulation Process between the Quantitative Results and the Emergent Qualitative Themes.

| Quantitative Domains | Triangulation Results | | Qualitative Themes | Sample Quote |
| --- | --- | --- | --- | --- |
| EORTC Physical Functioning* | | | | |
| Do you have any problem doing strenuous activity? | Agreement | | Daily Activities | “You’ll see the ironing pile and it’s getting bigger and bigger and bigger by the day and you think “I can’t get up to do it, I can’t stand long enough to do it” [Age 35, stage 1B, 3 months]. |
| Do you have any problem taking a long/short walk? | Agreement | | Walking | “I can’t walk as far…I’m frightened of falling” [Age 66 years stage 1B, 12 months].  “He’ll *(partner)* be like I’m taking the dogs for a long trek and I can’t go” [Age 35, stage 1B, 3 months]. |
|  | Complementary | | Lymphoedema | “My knees are twice the size they were…my ankles swell badly at night” [Age 66, stage 1B, 12 months]. |
|  | Complementary | | Incontinence | By the time I’d got to the second one *(stair)* I’m absolutely wet through” [Age 69, stage 2, 9 months]. |
|  | Complementary | | Drains | “It did start to get me down because I had to carry it *(drain)* around everywhere…..it got heavier and heavier as it filled up and I was just so slow, I was slow at doing things ” [Age 56, stage IB, 3 months]. |
| Do you need to stay in bed or chair all day? | Agreement | | Inactivity | “I got contented sitting down and watching television….I was sitting there all day watching all this rubbish” [Age 69, stage 2, 9 months]. |
| Do you need help washing/using toilet? | Agreement | | Help | “Obviously there were things that I couldn’t do like hoovering, shopping and things like that but I had help with that. I’ve got a sister and she does the shopping for me” [Age 38, stage 1B, 6 months]. |
|  | Complementary | | Numbness/lost sensation | I've lost all the feeling in between my legs [Age 69, stage 2, 9 months].  “I’ve still got numbness down where they took my lymph nodes out” [Age 37, stage IB, 6 months]. |
| EORTC Pain | | | | |
| ‘Have you had pain’? | Agreement | | Painkillers | “I’m reliant at the moment on painkillers” [Age 37, stage IB, 6 months]. |
|  | Complementary | | Site of Surgery | “It was basically pain from the site of the surgery ” [Age 66, stage 3A ,6 months]. |
|  | Complementary | | Extent of Pain | “The surgeon said "You’re going to be in a lot of pain because of this [operation]" but I didn't realise how much” [Age 66 years, stage 3A, 6 months]. |
|  | Complementary | | Lymphoedema | “There are certain nerves that are re-attaching so you can get shooting pains up one leg but for the rest of the time I just can’t feel my leg” [Age 37, stage IB, 6 months].  “It’s uncomfortable, it’s not painful and I can feel the legs swelling during the day, so when I’m at home and as much as possible I put the leg up” [Age 66, stage 3A, 6 months]. |
|  | Complementary | | Radiotherapy - Blisters | “And then I started to get blisters and I got about five or six blisters either side; now they were excruciating” [Age 56, stage IB, 3 months]. |
|  | Complementary | | Radiotherapy – Sunburn/burning | “Because it literally is *(skin)* burning, you know, it’s like you’re on fire, [Age 34, stage 4A, 3 months]. |
|  | Complementary | | Infection | “I got an infection and my stitches burst….as long as I took the painkillers and didn’t do too much I was fine” [Age 37, stage IB, 6 months]. |
|  | Complementary | | Stitches | “It was very uncomfortable with the stitches where I’d had the cut right on the edge, and that was catching a bit on my underwear” (Age 56, stage 1B, 3 months) |
|  | Complementary | | Tender/Sore/Bruising | “It was uncomfortable sitting down obviously because it was sore in that area” [Age 56, stage IB, 3 months]. |
| Did pain interfere with activities? | Agreement | | Pain and Daily Activities | “For the first two weeks, physically being able to walk was painful even going to toilet was extremely painful” [Age 37, stage IB, 6 months].  “I’m not as active because I’m in constant pain with my leg” [Age 66, stage 1B, 12 months]. |
|  | Complementary | | Pain and Intercourse | “We make love in other ways, not actually with penetrative sex, do you know what I mean, because it is still quite painful” [Age 42, stage IB, 6 months]. |
|  | Complementary | | Pain and Dilators | When I started using those dilators, that was quite excruciating but that got better” [Age 56, stage 1B, 3 months]. |
| EORTC Fatigue | | | | |
| Have you felt tired? | Agreement | | Tired | It’s slowed me down a hell of a lot. I can’t walk as far as I want to. I’m always tired. I’m constantly tired [Age 37, stage IB, 6 months]. |
|  | Complementary | | Role Functioning | “I’m still not on full duties, still on light duties at work so there’s things like carrying shopping in from the car and things like that I can’t do”. [Age 42, stage IB, 6 months]. |
|  | Complementary | | Tiredness Improving | “I‘m yawning again but saying that during the day I think I’ve got my old energy back” [Age 66, stage 3, 6 months]. |
| Have you needed to rest? | Agreement | | Apathy | “I had lost a lot of energy. I felt very drained” [Age 69, stage 2, 9 months]. |
| Have you felt weak? | Agreement | | Lethargy/Slow | “Sometimes I just wanted to lay down, you know…really lethargic and just wanted to lay down” [Age 34, stage 4A, 3 months]. |
| SF 36 Social Functioning | | | | |
| Has your physical condition or medical treatment interfered with your family life? | Agreement | | Impact upon Family | “It is really hard when you’re still very sore and tender and you say “Oh don’t jump on me. It was just a case of trying to love him *[eldest son]* but keep him at arms length” [Age 42, stage IB, 6 months]. |
|  | Complementary | | Communication | “I think the worst part of it, when you’ve been told you’ve got something like this is having to tell the rest of the family” [Age 66, stage 3A ,6 months]. |
| Has your physical condition or medical treatment interfered with your social activities? | Agreement | | Incontinence | “I can’t go out because if there’s no toilets, I’ve had it. I actually messed myself one time when I was on holiday, you can’t help it, can’t control it [Age 34, stage 4A, 3 months]. |
|  | Agreement | | Sitting | “I usually go out on a Saturday evening. I did stop that for about a month because of being uncomfortable. I didn’t want to go out to a pub or whatever, well it hurt [sitting down] and I couldn’t do it really, I don’t know, 4 or 5 weeks, I didn’t do that”. [Age 56, stage IB, 3 months]. |
|  | Complementary | | Confidence | That’s the worst bit that just got me down, really messed my confidence up, not being able to go out…not eating when I should, when I’m out” [Age 34, stage 4A, 3 months]. |
| ePAQ-PF General Sex Life | | | | |
| How much do sexual problems interfere with your enjoyment of life? | | Agreement | Impact of Sexual Problems on Daily Life | “I think I got the fear in my head. I was frightened. I thought “What if it doesn’t happen? What if it totally messes things up in life now? “Is it going to be OK? Will it work?” you know “I don’t know what they’ve done to me really” [Age 56, stage 1B, 3 months]. |
| Do you feel that you have lost interest in sex? | | Agreement | Lost Interest | “There’s no pressure, don’t get me wrong, but, no, I can’t, I couldn’t even entertain it now” (Age 69, stage 2, 9 months) |
| Overall, how satisfied are you with your sex life? | | Agreement | Not enjoyable | “I don’t find it probably as enjoyable as it was before” [Age 66, stage 3A ,6 months]. |
| Do problems with your health in general interfere with your sex life? | | Agreement | General health and sex life | “I don’t think there’ll be sex anymore, mind you at my age there shouldn’t be but having said that you do have feelings, you do get, you know, occasionally but I wouldn’t even dare to have it” [Age 56, stage 1B, 3 months]. |
| SF-36 Mental Health | | | | |
| Have you been a very nervous person? | Agreement | | Anxiety | “I was washing myself down below and I thought I could feel a lump again and I could feel myself getting panicky” [Age 51, stage 1B, 3 months]. |
|  | Complementary | | Fear of Recurrence | *“*The fear of not getting it all and for what’s going to happen to the children” [Age 42, stage IB, 6 months]. |
|  | Complementary | | Mortality | “I worry now and I think “What if I die first?” [Age 69, stage 2, 9 months].  “It will always be there that I’ve had it, cancer and I think that it will always come back and it will eventually take me away altogether” [Age 42, stage IB, 6 months]. |
|  | Complementary | | Viewing and Touching | “There was no way that I could look, just absolutely no way. And, when I’ve had a shower and I’m drying myself, I’ve found that I’m nervous” [Age 69, stage 2, 9 months]. |
| Have you felt so down in the dumps nothing could cheer you up? | Agreement | | Withdrawn | I basically withdrew into myself, I wouldn’t talk about it. I pushed everybody away that wanted to help” [Age 37, stage 1B, 6 months]. |
|  | Complementary | | Positivity | “ You have got to laugh about it and try not to dwell on it” [Age 66, stage 3A ,6 months]. |
| Have you felt calm and peaceful? | Agreement | | Mood Swings | “It’s been a very emotional roller coaster” [Age 42, stage IB, 6 months]. |
|  | Complementary | | Frustration | I think I got frustrated that I couldn’t do things and then when this drain would not stop emptying that upset me, I was [saying] I want it taking out because I can’t stand it anymore” [Age 56, stage 1B, 3 months]. |
| Have you felt downhearted and blue? | Agreement | | Depressed | “I used to cry at everything….burst into tears all the time” [Age 42, stage IB, 6 months]. |

*Due to the size of the table the SF-36 Physical Domain Questions (n = 10) have not been written in full but ‘agreement’ was observed with these items including: vigorous activities, such as running, lifting heavy objects, participating in strenuous sports, Moderate activities, such as moving a table, pushing a vacuum cleaner, bowling, or playing golf, lifting or carrying groceries, climbing, bending, walking, bathing/dressing.
